# Unleashing PD-1: a duel of immunity in aortic aneurysm formation

**DOI:** 10.1172/JCI182554

**Published:** 2024-08-01

**Authors:** Zhenguo Wang, Y. Eugene Chen, Lin Chang

**Affiliations:** Department of Internal Medicine, Frankel Cardiovascular Center, Michigan Medicine, University of Michigan, Ann Arbor, Michigan, USA.

## Abstract

Aortic aneurysms, particularly abdominal aortic aneurysms (AAAs), exhibit sex differences, with higher prevalence and severity in males than females, both in humans and experimental mouse models. In fact, male sex has been considered as the most potent nonmodifiable risk factor for AAA. Currently, there are no medications approved for the treatment of aortic aneurysms, despite the high lethality of ruptured aneurysms, which account for nearly 2% of all deaths. Moreover, the underlying molecular mechanisms mediating the sexual dimorphism of aortic aneurysms remain largely unknown. In this issue of the *JCI*, Mu et al. revealed a mechanism by which androgens, male sex hormones, exacerbate aortic aneurysms by suppressing programmed cell death protein 1 (PD-1) expression in T cells in an aldosterone and high salt–induced aortic aneurysm mouse model.

## Aortic aneurysms and sexual dimorphism

Aortic aneurysms (AAs) can occur in any segment of the aorta, including thoracic aortic aneurysms (TAAs) and abdominal aortic aneurysms (AAAs). AA typically remains asymptomatic until emergent, and lethal complications such as dissection or rupture occur (1). Ruptured AA has a mortality rate of over 80% before reaching the hospital (2), and almost 100% mortality within three days without rapid surgical or endovascular intervention (3). Therefore, early diagnosis and prevention of dissection/rupture are top priorities in managing this relatively rare but lethal aortic disease. To date, surgical repair is the primary treatment when AA rapidly enlarges, as no medications halt AA progression. Nevertheless, the overall in-hospital deaths related to postoperative complications remain high. Addressing critical-knowledge gaps in underlying mechanisms could substantially advance our understanding of AA pathophysiology and offer pivotal insights into nonsurgical treatments.

AA pathogenesis is complex and involves several underlying mechanisms. TAAs have a more robust genetic background than AAAs (4). Both TAAs and AAAs share common features, including aortic inflammation and the phenotypic switch and/or loss of vascular smooth muscle cells (VSMCs). Inflammation in AA involves the infiltration of inflammatory cells, including macrophages and T lymphocytes. CD4^+^ and CD8^+^ T cells, particularly CD4^+^ Th cells, drive aortic inflammation by releasing proinflammatory factors such as IFN-γ that promote chemotaxis of other inflammatory cells. These processes induce metalloproteinase expression, which contributes to AA formation and progression by promoting extracellular matrix degradation and VSMC phenotypic switching, ultimately weakening the aortic wall (5, 6).

Male sex has long been recognized as a potent nonmodifiable risk factor for AAA. AAA is approximately four to six times more prevalent in men than in women (7), but females experience faster rates of aneurysm growth, and their aneurysms tend to rupture at smaller sizes (8, 9). Yet there is limited understanding of how sex-specific differences in the aortic wall or sex hormones contribute to the development and progression of AA. Several studies have shown that estrogen protects against AA via reducing collagen destruction, remodeling, and immune cell migration (10). Consistently, AAA incidence is increased in postmenopausal women (11). This sexual dimorphism is also observed in experimental AAA models. Male mice infused with angiotensin II (Ang II) exhibited a four-fold higher prevalence of AAA compared with female mice. Interestingly, depletion of estrogen by ovariectomy does not influence AAA formation in female mice (12). In contrast, testosterone, a major androgen, promotes AAA incidence in male and female mice (12, 13), although epidemiologic studies suggest that men with AAA have lower serum testosterone compared with men without AAA (14). In hyperlipidemic mice (*ApoE^–/–^* mice), androgens mediate a higher incidence of Ang II–induced AAA through mechanisms that are independent of circulating renin or angiotensin receptor density. Instead, it increases the expression of the AT1a receptor, which was greater in the AAA-prone region of abdominal aortas of male but not female mice (12, 13). Moreover, androgen receptor (AR) in macrophages or VSMCs, but not in endothelial cells, mediates AAA development through IL-1α and TGF-β1 signaling (15). However, in normolipidemic C57BL/6J mice, testosterone depletion and AR blockade promote CaCl_2_ plus Ang II–induced AAA formation, which can be rescued by testosterone administration via regulating macrophage-mediated inflammatory responses (16). Androgens also suppress aortic lysyl oxidase activity, which is critical for the covalent cross-linking of collagen and elastin in mice (17). Nevertheless, the role of androgen in AA and the underlying molecular mechanisms remain to be fully investigated.

## Mechanisms of male hormone-promoting AAA formation

In this issue of the *JCI*, Mu and colleagues present compelling evidence that androgens exacerbate AA by suppressing programmed cell death protein 1 (PD-1) in mice (18). Using the aldosterone and high salt–induced (Aldo-salt–induced) AA model in ten-month-old mice (19), Mu and colleagues demonstrated that 70% of male mice, but none of the female mice, developed AAs. This incidence was dramatically reduced by androgen deprivation via orchiectomy and by downregulation or inhibition of AR by ASC-J9 or flutamide, respectively, while restoration of androgen by dihydrotestosterone pellet implantation reinstated aneurysms in orchiectomized mice (18). Androgen modulates gene expression by binding to and activating AR, a ligand-dependent transcription factor (20). Mu and colleagues identified 180 genes that were negatively responsive to androgen signaling in Aldo-salt–induced AA (18). Strikingly, these genes, including PD-1, were enriched by T cell receptor (TCR) signaling. PD-1 is an inhibitory receptor primarily expressed on activated T and B lymphocytes, macrophages, DCs, and monocytes, where it inhibits both adaptive and innate immune responses, mainly by binding to its ligand PD-L1 (21). Immunotherapies that target the PD-1/PD-L1 axis for cancer treatments have revolutionized this field. Furthermore, the PD-1/PD-L1 axis has been implicated in vascular inflammation and aneurysm (22, 23). Mu and colleagues further observed that splenectomy reduced Aldo-salt–induced AA. This reduction was accompanied by an enrichment of PD-1^+^ T cells and PD-1^+^ B cells in the aorta, probably originating from periaortic lymph nodes. Mechanistically, expression of PD-1 in the spleen was transcriptionally repressed by androgen-bound AR, which directly bound to the androgen response element in the promoter region of PD-1. Most notably, immune checkpoint blockade with anti–PD-1 antibody, adoptive PD-1–deficient T cell transfers, and genetic deletion of PD-1 all could reinstate Aldo-salt– or high-fat diet– and Ang II–induced AA (18). Additionally, Mu and colleagues identified IL-6 as an androgen-targeting gene in the aorta and found that inhibition of IL-6 signaling by LMT-28 partially blocked Aldo-salt–induced AA (18).

## Conclusions and implications

Hypertension is one of the most crucial risk factors for the development and dissection/rupture of AA. Although applications of Ang II receptor blockers showed limited protective effects on AAA progression in humans, Ang II infusion on hyperlipidemic mice is the most widely used mouse model worldwide for AA research (24). By using a previously established hypertensive model induced by Aldo-salt in aged mice (19), representing certain hypertensive populations, Mu and colleagues uncovered a mechanism by which androgens aggravate AA via the suppression of PD-1 expression in T cells within the spleen (18). This excellent study not only further emphasizes the potential of the Aldo-salt–induced AA model in the investigation of AA pathogenesis, but also provides clinical clues that immune checkpoint therapy in cancer treatment may adversely trigger the formation of AA in patients (25). Thus, imaging tests such as ultrasound for AA in patients with cancer undergoing such therapies may be promising in enhancing their health outcomes.

**Figure 1:**
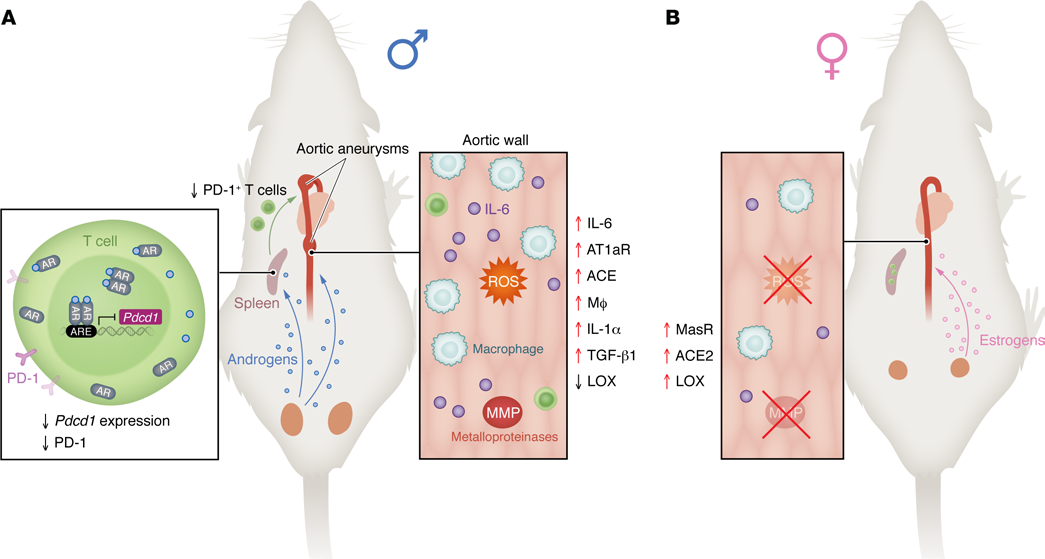
Sex hormones have differential effects on AA pathogenesis. Sex hormones have differential effects on aortic aneurysms pathogenesis. (**A**) Androgens promote the formation of AAA by increasing angiotensin II type 1a receptor (AT1aR), angiotensin-converting enzyme (ACE), IL-1α, and TGF-β1 signaling, as well as macrophage (Mφ) infiltration, and by suppressing aortic lysyl oxidase (LOX) activity. Mu et al. (18) identified a mechanism whereby androgen transcriptionally inhibits PD-1 gene expression in T cells within the spleen, rendering T cells into a less activated state. Meanwhile, the androgen induces IL-6 expression in the aorta. These changes promote the formation of aortic aneurysms in male mice. (**B**) Estrogens protect female mice from AAA pathogenesis by increasing LOX and ACE2 expression and promoting Mas receptor (MasR) signaling.

Considering the sexual dimorphism in AA (Figure 1), future basic and clinical research should aim to delineate specific factors responsible for region-specific aortic pathology, not only in the aortic wall but in the surrounding adventitial area, such as the perivascular adipose tissues, in males and females. Until now, most animal-associated therapeutic studies start interventions before or shortly after the AA induction, seeking to inhibit the onset or progression of AA, rather than to reverse established AA. However, the human clinical challenge is to reverse already established AA. Thus, future therapeutic research should prioritize strategies for reversing established AA. Meanwhile, efforts should be made to identify early serum or biopsy markers for AA.

## Version 1 08/01/2024

Electronic publication
